# Transdiagnostic App–Based Cognitive Bias Modification Intervention for Paranoia (Successful Treatment of Paranoia; STOP): Protocol for a Mixed Methods Process Evaluation Embedded in a Randomized Controlled Trial

**DOI:** 10.2196/81167

**Published:** 2025-12-22

**Authors:** Chloe Hampshire, Charlotte Dack, Laura Eid, Carolina Fialho, Alexandra Kenny, Hannah Scott, Rayan Taher, Theresa Taylor, Jenny Yiend, Pamela Jacobsen

**Affiliations:** 1 Department of Psychology University of Bath Bath United Kingdom; 2 Department of Psychosis Studies, Institute of Psychiatry, Psychology & Neuroscience King’s College London London United Kingdom; 3 McPin Foundation London, England United Kingdom

**Keywords:** paranoia, digital mental health, smartphone intervention, implementation, randomized controlled trial, process evaluation, implementation science

## Abstract

**Background:**

Paranoia (unfounded concerns that other people are deliberately trying to harm you) can be a distressing experience that impacts day-to-day functioning for many people. Digitally delivered interventions are a promising mode of treatment that can increase access to support and reach populations underserved by current provision. One such intervention is the Successful Treatment of Paranoia (STOP) intervention, which is a transdiagnostic self-administered smartphone app for paranoia that was evaluated in England for efficacy in a large, multisite randomized controlled trial. Although there is growing evidence regarding how STOP may work, little is known about the factors that influence its implementation during or after a trial. Understanding these factors is critical for supporting the future adoption of STOP and may inform the implementation of other digital mental health interventions.

**Objective:**

This paper aims to describe the protocol for a process evaluation that will explore intervention implementation to understand how much, by whom, and under what circumstances STOP was used in a trial context and what factors might influence future implementation in routine practice.

**Methods:**

We will conduct a mixed methods process evaluation informed by guidance published by the Medical Research Council in the United Kingdom and embedded in the main STOP efficacy randomized controlled trial, with the aim of understanding who agreed to try STOP, how they used STOP, and users’ and health care professionals’ views on factors that will affect future implementation, including barriers and facilitators. Process evaluation participants will include three samples: (1) STOP trial participants (randomized participants and nonrandomized referrals who agreed to try STOP), (2) individuals experiencing paranoia who participated in the Adult Psychiatric Morbidity Survey in England, and (3) health care professionals in England. We will use mixed methods data collected in the STOP trial (demographics, app use, recruitment data, and qualitative data exploring intervention acceptability from the perspectives of users and health care professionals) and demographic data from another paranoia population collected in the Adult Psychiatric Morbidity Survey.

**Results:**

The STOP trial was completed in December 2024, having recruited 274 participants. The process evaluation received funding in winter 2023. As of December 2025, data analysis for process evaluation studies 2 and 3 is ongoing following preregistration in July 2025. Results are expected to be completed and submitted for publication by February 2027.

**Conclusions:**

Findings from this process evaluation will help develop an evidence-based understanding of implementing STOP, including identifying suitable implementation strategies for posttrial delivery both inside and outside mental health services. Our findings will inform future iterations and the upscaling of STOP and may inform implementation of other self-administered digital mental health interventions.

**Trial Registration:**

International Standard Registered Clinical/Social Study Number ISRCTN17754650; https://www.isrctn.com/ISRCTN17754650

**International Registered Report Identifier (IRRID):**

DERR1-10.2196/81167

## Introduction

### Background

Paranoia refers to concerns that others may intend to harm you [1,2]. Freeman’s [1] theoretical model of paranoia suggests a hierarchy, whereby more severe forms of paranoid worries in clinical populations (eg, believing others are trying to kill you) build on milder, everyday forms of social evaluative concerns (eg, believing others are talking about you behind your back), which are commonly experienced in the general population. Paranoia is often a distressing experience that can negatively interfere with many aspects of life, including people’s everyday activities [3] and interpersonal relationships [4]. Many people can experience paranoia, including those with psychosis spectrum disorders, but it is also a transdiagnostic symptom and can be present in other mental health conditions as well, including depression and anxiety disorders [5]. Paranoia can also be experienced by nonclinical populations across the lifespan [3,6].

To improve the lives of those experiencing paranoia, developing support (eg, to reduce paranoia or sustain recovery) for this population has been identified as a research priority by patients, carers, researchers, and health care professionals in an international priority setting partnership [7]. One promising treatment mode for those experiencing paranoia is digital mental health interventions (DMHIs), or treatment that incorporates technology to improve users’ mental health [8]. An early value assessment conducted by the National Institute for Health and Care Excellence identified specific DMHIs as suitable for full recommendation, subject to further data collection [9]. Early recommendation of this sort can improve the provision of care by increasing treatment options and access to support using fewer resources than in-person treatments [9]. Preliminary evidence suggests that DMHIs may be an acceptable and feasible treatment for paranoia [10].

### What Is Successful Treatment of Paranoia?

While other DMHIs for paranoia have been developed, most are delivered via virtual reality, require clinical support, or focus on other psychotic experiences (eg, voices) and pay limited attention to more common, social evaluative concerns within paranoia (eg, that others are talking about you negatively) [10]. Successful Treatment of Paranoia (STOP) was developed to address these limitations by providing a self-delivered, smartphone-based transdiagnostic intervention specifically targeting interpretation biases underpinning paranoia. The intervention and its development have been described in detail elsewhere [11,12]. A brief description is included to contextualize our work.

STOP is a self-administered psychological intervention using the principles of cognitive bias modification for paranoia (CBM-pa). CBM-pa aims to address the cognitive interpretation bias that is thought to cause and maintain the symptoms of paranoia [13]. Individuals displaying this interpretation bias often interpret neutral or ambiguous social scenarios as personally threatening [13]. There is preliminary evidence from a feasibility randomized controlled trial (RCT) that interpretation biases can be modified by CBM-pa that was delivered via a desktop computer in a research laboratory setting (N=63) [14].

**Figure 1:**
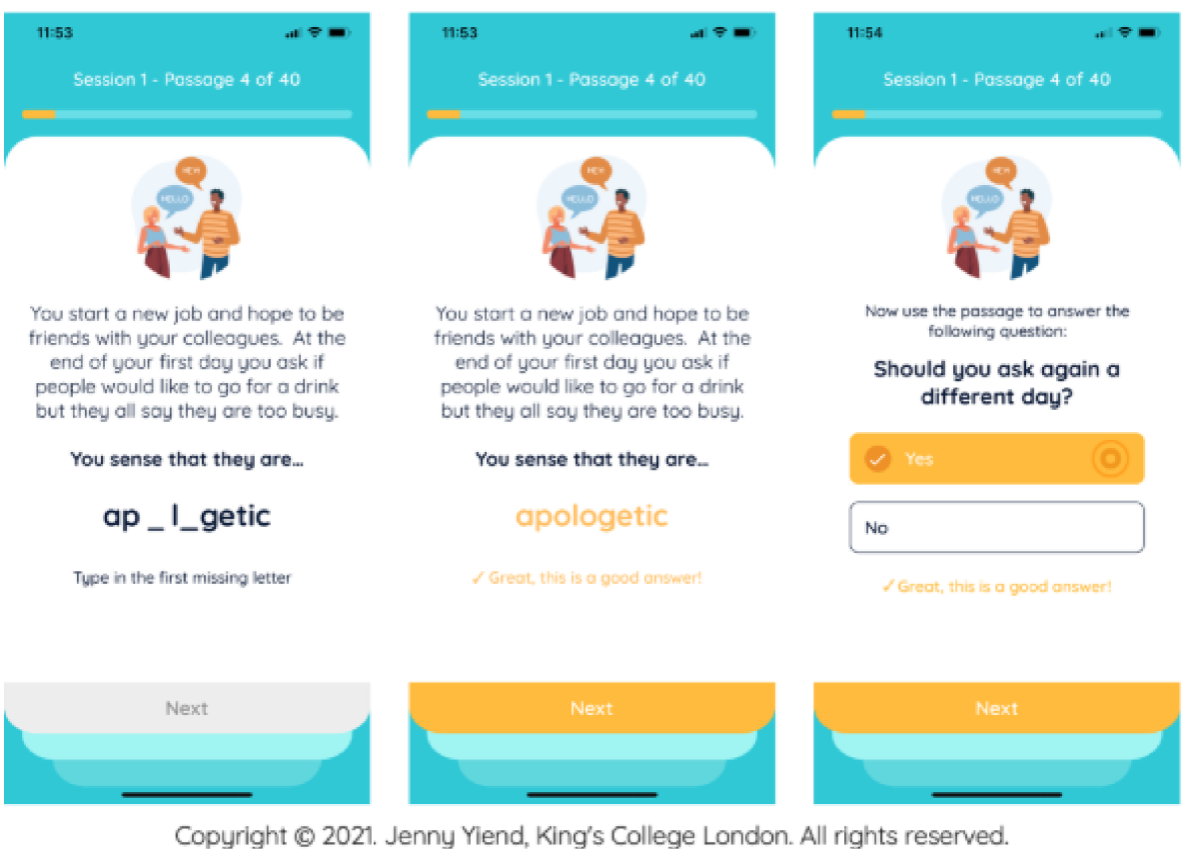
Successful Treatment of Paranoia (STOP) intervention example.

STOP in the RCT included a weekly session administered through a smartphone app either on users’ personal smartphones or, if needed to avoid digital exclusion, on a study phone with a data plan provided free of charge to participants. Two versions of the intervention of varying lengths were evaluated to investigate “dose” effects (6 vs 12 weeks). Users scheduled weekly sessions at convenient times, during which they read 40 everyday social scenarios. In the sessions, users were encouraged to interpret ambiguous social scenarios in a nonthreatening way by completing a word puzzle and then answering a question about the meaning of the scenario (Figure 1 includes an intervention example). Session content was designed by clinicians, academics, and the McPin Foundation research charity’s STOP lived experience advisory panel (LEAP) of 7 people who give voice to intended end users of the app to increase its clinical relevance and acceptability to users [11]. Automated phone and email reminders were sent at midnight 1 day before the scheduled session and again if a session was not completed on the scheduled day.

### How Do We Think STOP Will Work?

**Figure 2:**
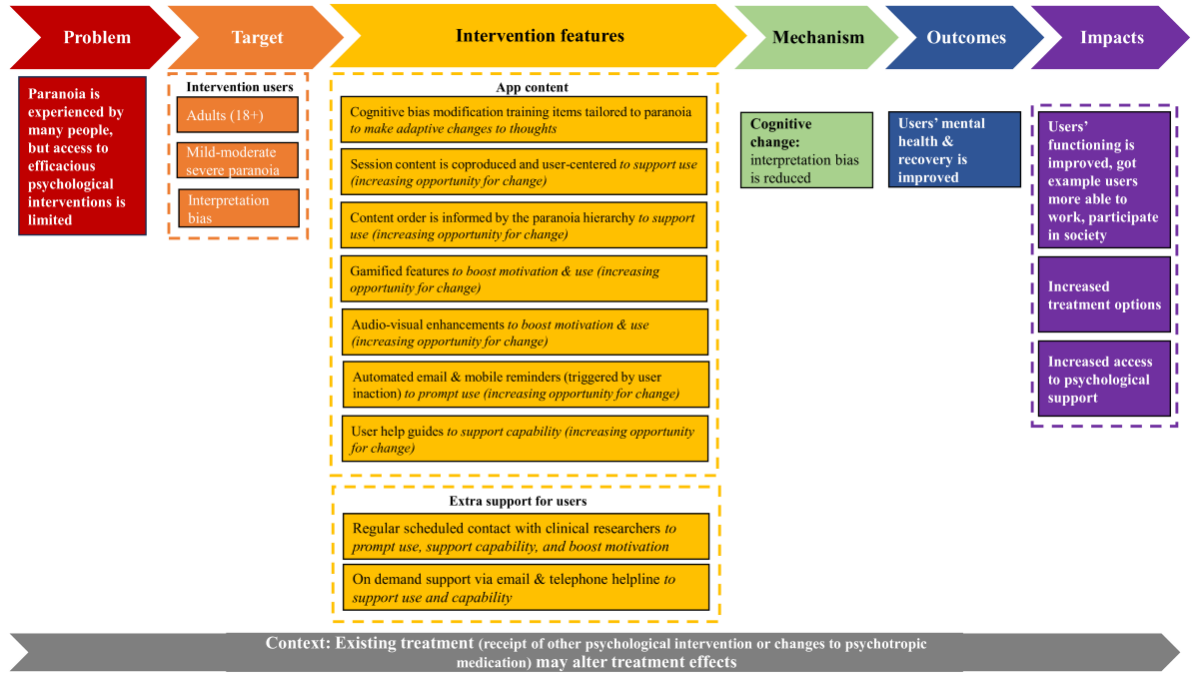
Logic model for the Successful Treatment of Paranoia (STOP) intervention.

Program theories underpin interventions and explain how, why, for whom, and in which circumstances interventions are assumed to work and can be represented visually in a logic model [15]. A logic model for the STOP intervention was created after conducting a document analysis of the trial protocol and publications associated with the intervention (Figure 2) [11,12]. An initial program theory was developed from the logic model (Textbox 1).

**Program theory for the Successful Treatment of Paranoia (STOP) intervention.**

- Users complete cognitive bias modification for paranoia (CBM-pa) training items delivered via a smartphone app, which creates a cognitive change that enables the user to consider a more neutral interpretation of ambiguous situations.
- The cognitive change has a positive impact on users’ mental health and recovery, including reducing symptoms of paranoia.

### Why Conduct a Process Evaluation?

We will conduct a process evaluation to better understand the factors influencing DMHI implementation both during and after the trial. Exploring factors that may influence implementation is an important research focus as the implementation of DMHIs is a persistent challenge [16,17], and it is also acknowledged that interventions may be used differently across settings and populations [18,19]. There is some empirical evidence for this as reviews have demonstrated DMHI use can vary due to several factors, including patient characteristics (eg, age) and intervention features (eg, including human support) [20]. However, implementation remains a key area of uncertainty in the STOP trial as it is currently unknown what, or if any, factors influence the implementation of STOP (Figure 2, context box). A limited understanding of implementing STOP contrasts with a comparatively better understanding of how STOP is assumed to work, for which there is a growing evidence base [13,14] and plans to evaluate mechanisms of action in other trial work [12]. Intervention implementation is an important uncertainty to address given that STOP has the potential to be implemented in a variety of settings (as users choose where and when sessions are accessed) and by a variety of potential end users (as users may not need to be under the care of a health service or have a formal mental health diagnosis to use STOP) [12].

### Evaluation Objectives

Our work aims to address these knowledge gaps by exploring the factors that influence the implementation of a self-administered digital intervention for paranoia in and after an RCT. The overarching objective of this evaluation is to develop an evidence-based understanding of implementing STOP by investigating how much, by whom, and under what circumstances STOP is and may be implemented in and after a trial. A secondary objective is to use this knowledge to identify the barriers and facilitators to implementing STOP in and after a trial.

## Methods

### Overview

We will conduct a mixed methods process evaluation of STOP. The protocol is reported using an amended version of the process evaluation recommendations from the Medical Research Council (MRC) process evaluation framework (Table S1 in Multimedia Appendix 1) [18]. We amended the reporting standards to fit the reporting of a protocol paper by removing sections that are not applicable (ie, those relating to reporting evaluation findings). Details of the core research team (CH, CD, JY, and PJ) are provided in Multimedia Appendix 2.

### Ethical Considerations

The STOP trial was preregistered at ISRCTN (ISRCTN17754650) and approved by the London-Stanmore Research Ethics Committee (21/LO/0896, January 14, 2022) and the Medicines and Healthcare products Regulatory Agency (CI/2022/0033/GB, July 19, 2022), including approval for collecting additional qualitative data with STOP users and health care professionals. An additional qualitative study involving STOP trial participants was conducted and approved separately by the University of Bath Social Sciences Research Ethics Committee (0179-353). Written informed consent was obtained from all participants prior to data collection, with the option to withdraw at any time without specifying a reason. Participants were compensated for their time: trial participants received up to £115 (US $153; £20 [US $26.6] per follow-up assessment, an additional £20 [US $26.6] at the trial end, and £15 [US $19.95] after completing an additional interview), health care professionals received a £20 (US $26.6) voucher, and nonrandomized STOP users received £40 (US $53.2). All participants were assigned unique study identifiers, and only fully anonymized and deidentified data will be accessed for analyses. The process evaluation was approved by the University of Bath Social Sciences Research Ethics Committee as a requirement of the doctorate (5841-6196). Further ethics approval was not required because the process evaluation did not introduce additional trial procedures or data collection and will only use existing data from participants who consented to data archiving.

### Process Evaluation Design

We will use the 2015 MRC process evaluation guidance as a methodological framework, which assumes that an efficacy evaluation in isolation provides a limited understanding of an intervention [18]. Instead of evaluating intervention efficacy, process evaluation in the MRC framework explores 3 proximal outcomes which can complement efficacy evaluations (Textbox 2) [18].

**Medical Research Council (MRC) process evaluation components.**

- Context: external factors that influence intervention functioning or delivery.
- Implementation: intervention delivery in the evaluation.
- Mechanisms of impact: how interventions produce change.

### How is STOP Being Evaluated?

#### Efficacy RCT

The process evaluation is embedded within the host RCT evaluating the efficacy and safety of STOP compared with an active control condition (a 12-week text reading control delivered through the STOP app, where users read and responded to items, including facts or unambiguous everyday experiences without a social component, such as making a cup of coffee) [12]. The RCT was preregistered (ISRCTN17754650), and a detailed protocol has been published [12]. The RCT was run by 2 main recruitment coordinating hubs at King’s College London and the University of Bath in England [12]. Recruitment in the RCT ran from September 2022 to June 2024, closing after 274 participants were randomized, and data collection was completed in December 2024. Participants were recruited from 3 main sources, including 20 National Health Service (NHS) trusts, third sector organizations coordinated by the McPin Foundation, and via self-referral routes using recruitment posters or the study website [12]. Individuals aged 18 years or older who were experiencing mild to moderate-severe paranoia (operationalized as a score between 3 and 6 out of 7 on item 6 on the Positive and Negative Syndrome Scale [21] during screening) and displaying an interpretation bias toward paranoid meanings (operationalized as a score between −2 to +3 on a scale ranging from −3 to +3 the Similarity Rating Task during screening) were eligible for the RCT [12]. Individuals who were at high risk of suicide, lacked the capacity to consent, or could not communicate in written or spoken English were excluded from the RCT [12].

#### Planned Process Evaluation Studies

**Figure 3:**
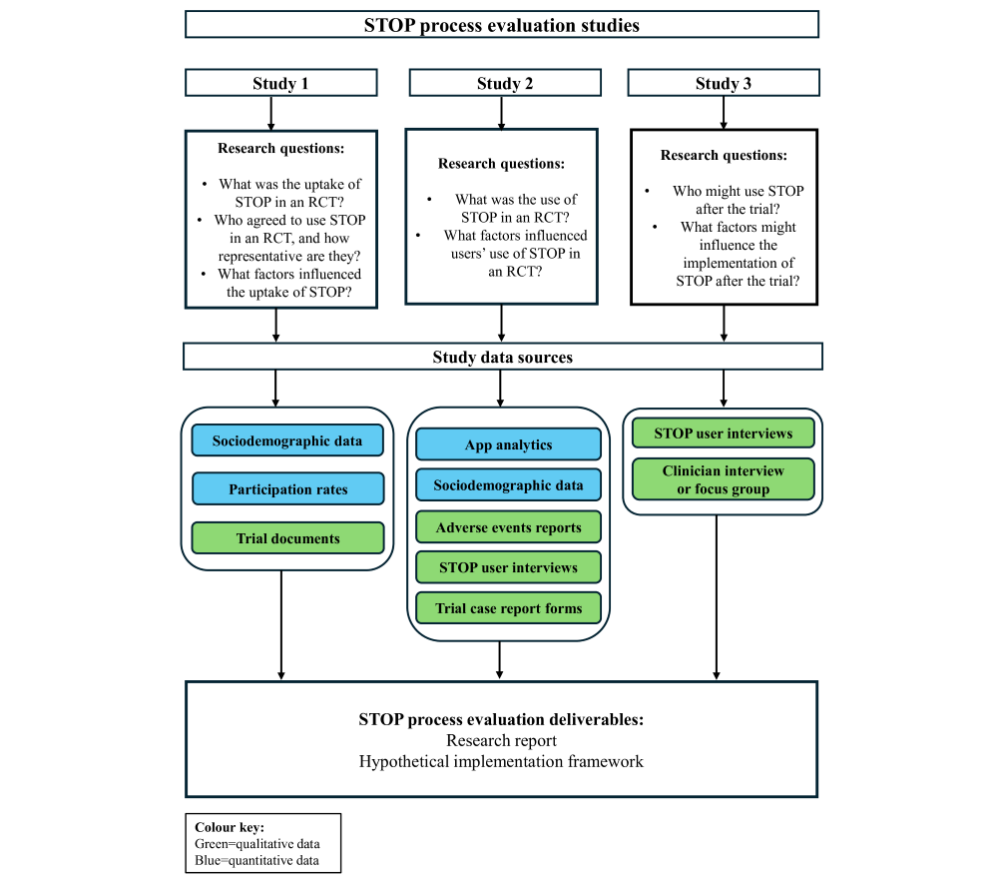
Overview of Successful Treatment of Paranoia (STOP) process evaluation questions and methods. RCT: randomized controlled trial.

We will conduct 3 studies that will explore the implementation of STOP. The data sources and research questions per study are summarized in Figure 3. Study methods and progress are reported for each of the planned studies in further detail below. The process evaluation is preregistered on the Open Science Framework [22].

#### Study 1: Exploring the Uptake of STOP in an RCT

### Overview

This mixed methods study will conduct analyses on STOP trial routine data and secondary analyses on sociodemographic data from another paranoia population in England.

### Aim

To summarize who agreed to take part and try STOP in a trial (STOP uptake), to describe factors that influenced uptake, and to characterize and assess the representativeness of those agreeing to try STOP compared with another paranoia population in England.

### Participants and Recruitment

STOP RCT participants and recruitment procedures are described earlier (in the Efficacy RCT section) and in full in the protocol paper [12].

### Measures

Measures will include intervention uptake, factors influencing uptake, and the characteristics of individuals who agreed to try STOP compared with a paranoia population in England. Intervention uptake will be summarized using trial records to report the number and percentage of those agreeing to try STOP out of those who were approached to take part in the trial at different time points in the RCT (eg, consent, screening, and randomization) to summarize the uptake of STOP in a trial.

Factors influencing uptake will be identified from qualitative reasons reported by those agreeing to and declining to try STOP at different time points in the RCT (eg, consent, screening, and randomization), using STOP trial documents (eg, CONSORT diagrams, STOP case report forms, and recruitment trackers) to describe factors that facilitated or hindered the uptake of STOP in a trial.

Characteristics of those agreeing to try STOP compared with a paranoia population in England will be assessed using sociodemographic data obtained from STOP trial baseline data and from the Adult Psychiatric Morbidity Survey (AMPS) conducted in 2023 to 2024 by Public Health England, which provides data about treated and untreated psychiatric disorders among adults in England [23], to characterize and assess the representativeness of those agreeing to try STOP. We will compare those who agreed to try STOP with the subsample of responders endorsing items indicating paranoid thinking, which is a method that has been adopted in other analyses of AMPS [24,25]. We are unable to prespecify the variables or which items and measures will be used to indicate paranoia because data from the AMPS are not available at the time of publication [23]. However, based on the most recently available AMPS dataset published in 2016 [26], probable variables include age, sex, ethnicity, living arrangement, region, employment status, and current treatment.

### Analysis

Rates of uptake will be summarized descriptively. Qualitative data from STOP trial documents will be analyzed in the latest version of NVivo (Lumivero) to provide an audit trail using a framework analysis approach [27,28], and findings from all trial documents will be triangulated. We will use the Consolidated Framework for Implementation Research (CFIR) [29] as a deductive framework to identify factors influencing uptake. Data that cannot be coded using a priori codes from the framework will be inductively coded in an “other” category. Qualitative analysis will be led by CH, but codes and themes will be developed and reviewed by the core research team (CD, JY, and PJ). STOP trial participants will be characterized using descriptive statistics from the trial’s main outcome paper, supplemented as necessary by further information. Exploratory quantitative analyses will be conducted using relevant packages in the most recent version of R software (R Foundation for Statistical Computing) to test for between-group differences using chi-square tests and 2-tailed *t* tests where appropriate. If necessary, a *P* value correction will be used to account for multiple testing.

### Status

Data collection is complete, but analysis has not yet started.

#### Study 2: Exploring How Much STOP Was Used in an RCT, and by Whom

### Overview

This mixed methods study will use STOP trial routine data and will conduct secondary analyses of qualitative data generated from interviews with STOP users who consented to data archiving. The interviews were originally conducted as part of a qualitative evaluation of the host STOP trial or other research work, and not as part of the planned process evaluation, and have been previously disseminated as peer-reviewed publications [30,31].

### Aims

To summarize how much STOP was used in an RCT, to characterize users of STOP, and to describe factors that predicted or influenced its use.

### Participants

Participants include 182 trial participants who were randomized to the intervention arms, and 10 STOP intervention users consenting to participate in interviews for data archiving. The eligibility criteria for trial participants are described earlier (in the Efficacy RCT section). No exclusion criteria were added to participate in the interview studies, although STOP users needed an email account and access to a phone or computer to take part [30,31]. Interviewees were predominantly female (15/25, 60%) and White (16/25, 64%) [30].

### Recruitment

The recruitment procedure for the host STOP trial is described earlier (in the Efficacy RCT section). In the qualitative studies, 2 samples participated in a one-off interview. The first sample comprises 8 participants who had completed the host STOP trial, were randomized to an intervention arm, provided informed consent for an optional qualitative interview, and consented to data archiving [30]. The second sample included 12 participants who were ineligible for the host trial but had tested a 6-week version of the STOP intervention on a dedicated study phone and consented to a one-off interview; the 2 participants who consented to data archiving will be included in our analyses [31].

### Measures

Measures will include intervention use, predictors of use, and factors that hindered or facilitated use. Intervention use in the trial is the primary outcome of study 2 and will be quantified and treated as a count variable based on the number of sessions completed (0-6 or 0-12) for 6 or 12 weeks, which was electronically monitored by a digital health platform (HealthMachine; Avegen Health). Acceptable and target session completion was defined in the study protocol as the user entering a response on 20 (50%) or 30 (75%) training items per session, and both completion rates will be considered.

Predictors of use will be selected from 10 quantitative variables in STOP trial routine baseline data. Our selection was informed by available trial data and an existing digital engagement model [32]. The digital complexity model proposes a person-centered approach to treatment and is informed by established models of engagement [32], such as the cumulative complexity model [33] and has been adopted in other evaluations [34]. Predictors include user variables (life demands, resources, and clinical characteristics) and STOP intervention variables (delivery device and therapy burden). User life demands include self-reported employment and marital status. User clinical characteristics include self-reported number of years living with paranoia, self-reported paranoia severity measured using a validated clinical measure (the Paranoia Worries Questionnaire) [35], and interpretation bias severity measured using the Similarity Rating Task. We selected the most sensitive measures of paranoia and interpretation bias severity as predictors after observing data in the host trial. User resources include self-reported dyslexia and premorbid IQ measured using the Wechsler Test of Adult Reading [36]. Intervention components include the STOP delivery device (users’ own smartphone or a study-provided phone) and intervention length (6 or 12 weeks, based on the allocated trial arm), both recorded in electronic databases used for the trial (MACRO; Ennov and HealthMachine).

Barriers to intervention use will be identified from qualitative self-reported reasons for lack of use from 3 sources. First, users reported reasons for missed sessions during check-in calls with a trained researcher. Second, reasons for withdrawal from the host trial are recorded in an electronic database (MACRO). Third, pooled interviews with STOP users will be used to identify whether any factors that hindered use were reported outside of routine trial data.

Facilitators to intervention use will also be identified from pooled interviews with STOP to determine whether any factors that facilitated intervention use were reported outside of routine trial data.

### Procedure: Qualitative Interviews

A semistructured topic guide was created for each qualitative study (Supplementary Materials S1 and S2 in Multimedia Appendix 3). The topic guides were developed to explore users’ experiences of a digital therapeutic relationship [30] and the perceived acceptability of STOP [37]. Trained researchers (TT and LE) conducted all one-off interviews online, which were recorded and transcribed. Anonymized transcripts will be accessed from secure institutional servers.

### Analysis

Quantitative analyses will be conducted using relevant packages in the most recent version of R. To contextualize the substudy findings, STOP users will be characterized using descriptive statistics, including clinical and sociodemographic variables, taken from the trial’s main outcome paper and supplemented as necessary by further information, and reported per intervention arm. Chi-square tests or pairwise comparisons will be used to test for differences between groups. To report intervention use, descriptive statistics will summarize the total number and proportion of intervention users in the host trial completing 0 to 6 or 0 to 12 sessions using acceptable and target rates. To test predictors of engagement, we will first assess the nature of the distribution and then run a linear regression analysis for normally distributed data or Poisson or negative binomial regression if data are not normally distributed (depending on overdispersion in the data). We will qualitatively analyze self-reported barriers and facilitators to intervention use using the same approach in study 1 (in the Analysis section) as analysis will be led by CH, supported by NVivo, and will deductively use the CFIR framework [29] in framework analysis [27,28], while inductively coding other data not captured in the initial framework.

### Status

Data collection is complete, but analyses have not yet started.

#### Study 3: Qualitatively Exploring the Hypothetical Posttrial Implementation of STOP

### Overview

This qualitative study conducted interviews and focus groups with health care professionals and will conduct secondary analyses on interviews with STOP users (described under study 2).

### Aim

To identify the hypothetical barriers and facilitators to implementing STOP after the trial using the perspectives of STOP users and health care professionals.

### Participants

Participants include STOP users and health care professionals. The STOP user participants are described previously (in the Participants section for study 2). Twenty health care professionals from 6 NHS trusts in England participated in an interview (n=11) or focus group (n=2). These participants were aged 26 to 61 (mean 38, SD 11.736) years and were predominantly clinical psychologists (15/20, 75%), female (15/20, 75%) and White (16/20, 80%).

### Recruitment

The recruitment processes for STOP users are described earlier (in the Recruitment section for study 2). Health care professionals were recruited from NHS trusts in England. Participants were those who provided informed consent to take part in a one-off interview or focus group after trialing a tester version of the STOP intervention or had viewed a video demonstrating the app.

### Procedure

The procedure for the STOP user interviews has been described previously (in the Procedure section for study 2). The health care professional topic guide was developed to explore the perceived acceptability of STOP (Supplementary Material S3 and S4 in Multimedia Appendix 3). Trained researchers (CH, AK, and HS) conducted the interviews and focus groups using Microsoft Teams, which were recorded and transcribed.

### Analysis

Descriptive statistics for anonymized basic demographic data will be tabulated. To date, interviews and focus groups have been transcribed verbatim. Next, we will qualitatively analyze data using the same approach in study 1 (in the Analysis section) with analysis led by CH, supported by NVivo, and deductively using the CFIR framework [29] in framework analysis [27,28], while inductively coding other data not captured in the initial framework.

### Status

Data collection and transcription are complete, and preliminary analyses have started.

#### Integrating Qualitative and Quantitative Data

Integration of quantitative and qualitative findings will be undertaken using a triangulation approach guided by the CFIR framework [29]. Integration will occur at the interpretation and reporting stages through a joint comparison of quantitative indicators (eg, predictors) and qualitative themes on barriers and facilitators. Quantitative and qualitative data will be synthesized within the CFIR framework [29] to identify key barriers and facilitators to implementing STOP in and after a trial (process evaluation aim 2). Therefore, our process will identify convergent and divergent findings and provide an evidence-based understanding of factors influencing implementation using mixed methods data.

#### Patient and Public Involvement

Patient and public involvement in the STOP trial is contributed via the LEAP, coordinated by the McPin Foundation, and is described in more detail in the protocol paper [12]. Within the process evaluation, the LEAP contributed to developing the health care professional topic guide, provided input on the process evaluation design, and a lived experience peer researcher collected and transcribed data in study 3.

#### Process Evaluation Deliverables and Dissemination Plan

We expect 2 deliverables from the process evaluation: (1) a research report and (2) a hypothetical implementation framework. First, we will report the process evaluation in an implementation paper. Second, our findings will generate a hypothetical implementation framework for the delivery of STOP after the trial, which will include implementation strategies that are tailored to delivering STOP both inside and outside mental health services. Both deliverables will be reviewed by the STOP LEAP, and disseminated as peer-reviewed, open-access journal articles and conference presentations. Our work will also be disseminated to potential end users through existing connections via the McPin Foundation’s networks, the STOP LEAP, and other relevant end user networks.

## Results

The STOP trial is now complete, including recruitment, data collection, and primary outcome analyses. Data collection concluded in December 2024, with 274 participants recruited. The primary outcome paper for the STOP trial is expected to be submitted for publication in winter 2025. Data collection for data used in the process evaluation was also completed in January 2025, and all data have been cleaned. Process evaluation analyses began on study 3 in August 2025 following preregistration in July 2025 and are expected to be finalized in winter 2025. Analyses for process evaluation studies 2 and 1 will commence in winter 2025 and spring 2026, respectively. Results from all 3 studies are expected to be finalized and submitted for publication in 2026. The overall process evaluation, including the development of a posttrial implementation framework, will be completed by February 2027.

## Discussion

### Expected Findings

Our process evaluation is expected to provide a detailed understanding of factors that influence implementation within a trial context. An evidence-based understanding of implementing STOP in an RCT will address a key uncertainty in the STOP logic model (Figure 2) and provide a more accurate understanding of actual implementation processes, which may differ from hypothesized ones [38]. Our process evaluation therefore offers unique insights beyond other STOP trial projects and can complement and contextualize efficacy findings. Insights into intervention-related implementation barriers and facilitators will inform future iterations of STOP. We will also develop an implementation framework for posttrial delivery of STOP, which will inform the upscaling of the intervention outside of an RCT setting; a persistent challenge [16,17] that must be addressed to avoid underuse of research efforts and to ensure the provision of optimal care to those experiencing mental health difficulties. Our framework may also be useful for other researchers or clinicians seeking to integrate self-administered DMHIs into routine care.

### Comparison to Prior Work

Our findings can address important gaps in research and knowledge in this field. The process evaluation is conducted in a context where there is interest in developing treatments for paranoia [7], including those that are digitally delivered [2]. While an oft-cited advantage of digital treatments is the potential to implement this mode in a variety of settings [39], the literature has focused on service settings [38,40,41], which often have limited access to the resources needed to implement DMHIs [42]. Therefore, we will address this limited opportunity to learn about DMHI implementation in other settings, including settings without clinical support or guidelines, which may be of interest to other evaluators in this field. Furthermore, the process evaluation methodology is often underutilized [43,44]. Our work therefore addresses a methodological gap and aligns with wider priorities to strengthen implementation research in digital mental health [16,45] and may add to the growing evidence base that a concurrent investigation of efficacy and implementation using process evaluation can improve intervention evaluation and development in trials [18,46]. Finally, an evidence-based understanding about implementing STOP has implications for the provision of care to those experiencing paranoia. Our work could be applied to a care context where health care professionals can assume that those with paranoia are unsuitable for this treatment mode due to patients’ illness factors, such as preexisting concerns about technology [47,48]. Evidence suggests that such staff views can be overgeneralizing, as paranoia does not preclude engagement with DMHIs [10] or can coexist with their use [49].

### Strengths and Limitations

This process evaluation has several strengths. First, to our knowledge, we are the first to explicitly apply process evaluation methodology to evaluate a DMHI used by a transdiagnostic sample of individuals with paranoia. Furthermore, we undertake a rigorous approach to process evaluation by adopting an established framework for this evaluation method [18]. An additional strength is that most process evaluation data were collected as part of a large-scale clinical trial and thus were subject to quality control checks and may be less subject to responder bias, meaning that more reliable data will be used. Furthermore, we will use mixed methods data and a range of data sources, which may identify different factors affecting implementation. Triangulation of mixed methods data in analyses will enable an integrated interpretation of process evaluation findings, which will support the development of an evidence-based understanding of implementation that may be used in future work. Finally, we transparently report on the planned process evaluation approach, scope, and methods to contribute to good practice and share our work with other stakeholders.

However, the process evaluation should be considered in line with several limitations. Uptake rates may be limited by incomplete recording of individuals who declined participation at the initial approach, as data were only available for contacts made directly by STOP research assistants or shared with the trial team. Our use of secondary interview data with STOP users has several limitations. These studies did not explicitly address our evaluation aims, which may have limited the depth or relevance of feedback from STOP users to our process evaluation. Furthermore, the use of secondary data meant that we were unable to analyze data during data collection, limiting our ability to explore emergent themes. Qualitative data were also collected using opportunity sampling, which may introduce bias toward responders who may be more interested in intervention evaluation or had more positive experiences of STOP. Future evaluation work in the field could mitigate this limitation by prospectively embedding a process evaluation within trials. Additionally, we could not prespecify which variables we will use to assess sample representativeness in study 1 as the AMPS protocol or data are not available at the time of publication. Finally, the protocol will be published after data collection was completed for the process evaluation studies. To mitigate these limitations and enhance transparency, we will prespecify our analysis plans on Open Science Framework before starting analyses [22].

### Conclusions

Overall, our work will develop an evidence-based understanding of the implementation of STOP in and after a trial, including identifying barriers to and facilitators of its implementation. Our process evaluation is also expected to develop a hypothetical implementation framework for posttrial delivery inside and outside of mental health services, which will inform upscaling and future iterations of the STOP intervention. Expected findings are anticipated to also have implications beyond the STOP trial and intervention. Our findings may also highlight the importance of process evaluation when trialing interventions by generating evidence that can inform the development or upscaling of other self-administered DMHIs.

## Appendix

Multimedia Appendix 1 Process evaluation reporting standards completed checklists.

Multimedia Appendix 2 Process evaluation team expertise.

Multimedia Appendix 3 Interview and focus group topic guides.

## Abbreviations

AMPS: Adult Psychiatric Morbidity Survey
CBM-pa: cognitive bias modification for paranoia
CFIR: Consolidated Framework for Implementation Research
DMHI: digital mental health intervention
LEAP: lived experience advisory panel
MRC: Medical Research Council
NHS: National Health Service
RCT: randomized controlled trial
STOP: Successful Treatment of Paranoia
