# Supplementary material for: Transdiagnostic App–Based Cognitive Bias Modification Intervention for Paranoia (Successful Treatment of Paranoia; STOP): Protocol for a Mixed Methods Process Evaluation Embedded in a Randomized Controlled Trial
Source: JMIR Res Protoc. 2025 Dec 22;14:e81167. doi: 10.2196/81167 (PMC12770924; doi:10.2196/81167)
Supplement: Multimedia Appendix 1 [file resprot_v14i1e81167_app1.docx]

**Multimedia Appendix 1: Supplementary Materials**

# Supplementary Table S1. Medical Research Council (MRC) reporting standards for realist process evaluations (Moore et al., 2015).

| **TITLE** | | **Reported in document**  **Y/N/Unclear/NA** | **Page(s) in document** |
| --- | --- | --- | --- |
| PLANNING | | | |
| 1 | Carefully define the parameters of relationships with intervention developers or implementers   - Balance the need for sufficiently good working relationships to allow close observation, against the need to remain credible as independent evaluators - Agree whether evaluators will take an active role in communicating findings as they emerge (and helping correct implementation challenges) or have a more passive role | Y | Multimedia Appendix 2, Supplementary Material 1 |
| 2 | Ensure that the research team has the correct expertise. This may require:   - Expertise in qualitative and quantitative research methods - Appropriate interdisciplinary theoretical expertise | Y | Multimedia Appendix 2, Supplementary Material 1 |
| 3 | Decide the degree of separation or integration between process and outcome evaluation teams   - Ensure effective oversight by a principal investigator who values all evaluation components - Develop good communication systems to minimise duplication and conflict between process and outcomes evaluations Ensure that plans for integration of process and outcome data are agreed from the outset | Y | Multimedia Appendix 2, Supplementary Material 1 |
| DESIGN & CONDUCT | | | |
| 4 | Clearly describe the intervention and clarify causal assumptions (in relation to how it will be implemented, and the mechanisms through which it will produce change, in a specific context). | Y | 4-6,  Figures 1 & 2 |
| 5 | Identify key uncertainties and systematically select the most important questions to address. | Y | 6-8 |
| 6 | Select a combination of methods appropriate to the research questions | Y | 10-19, Figure 3 |
| PLANNED ANALYSES | | | |
| 7 | Consider more detailed modelling of variations between participants or sites in terms of factors such as fidelity or reach (eg, are there socioeconomic biases in who received the intervention?) | Y | 11-16 |
| 8 | Integrate quantitative process data into outcomes datasets to examine whether effects differ by implementation or prespecified contextual moderators, and test hypothesised mediators | N/A | Addressed in other evaluation work |
| 9 | Collect and analyse qualitative data iteratively so that themes that emerge in early interviews can be explored in later ones | N | Addressed 22 |
| 10 | Ensure that quantitative and qualitative analyses build upon one another (eg, qualitative data used to explain quantitative findings or quantitative data used to test hypotheses generated by qualitative data) | Y | Figure 3 |
| 11 | Where possible, initially analyse and report process data before trial outcomes are known to avoid biased interpretation | Y | Addressed multimedia Appendix 2, Supplementary Material 1 |
| 12 | Transparently report whether process data are being used to generate hypotheses (analysis blind to trial outcomes), or for post-hoc explanation (analysis after trial outcomes are known) | Y | Addressed multimedia Appendix 2, Supplementary Material 1 |
| REPORTING | | | |
| 13 | Identify existing reporting guidance specific to the methods adopted | Y | 8-9 |
| 14 | Report the logic model or intervention theory and clarify how it was used to guide selection of research questions and methods | Y | 6-7,  Figure 2 |
| 15 | Disseminate findings to policy and practice stakeholders | Y | Addressed 18 |

Reproduced from: Moore, G. F., Audrey, S., Barker, M., Bond, L., Bonell, C., Hardeman, W., ... & Baird, J. (2015). Process evaluation of complex interventions: Medical Research Council guidance. *bmj*, *350*.
