# Supplementary material for: Transdiagnostic App–Based Cognitive Bias Modification Intervention for Paranoia (Successful Treatment of Paranoia; STOP): Protocol for a Mixed Methods Process Evaluation Embedded in a Randomized Controlled Trial
Source: JMIR Res Protoc. 2025 Dec 22;14:e81167. doi: 10.2196/81167 (PMC12770924; doi:10.2196/81167)
Supplement: Multimedia Appendix 2 [file resprot_v14i1e81167_app2.docx]

**Multimedia Appendix 2: Supplementary Materials**

Supplementary Methods SM1. Process evaluation team expertise.

The process evaluation research team has expertise in qualitative and quantitative methods. We are an interdisciplinary team, and leverage expertise from two institutions in the England (University of Bath and King’s College London). CH worked as a trained researcher on the host trial for three years, and thus has strong working knowledge of the evaluation. CH also has experience of qualitative methods and clinical psychology and has undertaken training in quantitative methodology and implementation science, including process evaluation methodology. CD has expertise in health psychology, qualitative methods and implementation science. PJ is a clinical psychologist, with experience working with those with psychosis including paranoia, and expertise in quantitative research methods, and was a principal investigator on the host trial. JY has extensive expertise in cognitive psychology, quantitative methods, and intervention development. JY is the chief investigator of the host randomised controlled trial, with oversight of the evaluation of intervention efficacy, and supports the proposed additional process evaluation work, planned work does not duplicate or conflict with the original outcome evaluation. We have established and will continue to use communication systems between the process evaluation team and efficacy evaluation team to prevent duplication and/or conflict between evaluation teams. As data collection has completed in the host trial, it is not possible to communicate findings as they emerge or to use process evaluation data to correct trial implementation issues. However, process evaluation analyses will be completed independently from other evaluation work in the trial, though JY will have oversight of all projects and will communicate or coordinate findings as appropriate.
