# Supplementary material for: Transdiagnostic App–Based Cognitive Bias Modification Intervention for Paranoia (Successful Treatment of Paranoia; STOP): Protocol for a Mixed Methods Process Evaluation Embedded in a Randomized Controlled Trial
Source: JMIR Res Protoc. 2025 Dec 22;14:e81167. doi: 10.2196/81167 (PMC12770924; doi:10.2196/81167)
Supplement: Multimedia Appendix 3 [file resprot_v14i1e81167_app3.docx]

**Multimedia Appendix 2: Supplementary Materials**

Contents

[Supplementary Methods SM1. Semi-structured interview topic guide on the perceived acceptability of STOP (Successful Treatment of Paranoia) – STOP app users. 2](#_Toc194577740)

[Supplementary Methods SM2. Semi-structured interview topic guide on digital therapeutic alliance – STOP (Successful Treatment of Paranoia) app users. 4](#_Toc194577741)

[Supplementary Methods SM3. Semi-structured focus group topic guide on the perceived acceptability of STOP (Successful Treatment of Paranoia) – mental health workers. 6](#_Toc194577742)

[Supplementary Methods SM4. Semi-structured focus interview topic guide on the perceived acceptability of STOP (Successful Treatment of Paranoia) – mental health workers. 10](#_Toc194577743)

# Supplementary Methods SM1. Semi-structured interview topic guide on the perceived acceptability of STOP (Successful Treatment of Paranoia) – STOP app users.

**User Semi-Structured Interview Topic Guide**

*(Numbered questions are used to structure the interview. Bulleted questions are used only as prompts if needed to trigger more discussion.)*

1 . **How did you find using STOP: if it had been a prescribed therapy, would you have been satisfied with it?**

- Consider its content, interface, menus, calendar, reminders, colour scheme, branding/logo, badges, trivia etc.
- What was the most/ the least satisfactory thing?
- How do you think STOP could be improved?

2. **How did you find the visual and audio features of STOP (the pictures and sounds that went along with each item)?**

- How clear or unclear were the pictures and text?
- What did you like most/ least about the way things were presented in the app?
- How would you improve the look and feel of STOP?

3. **What did you think of the scenarios presented in the STOP mobile app?**

- How easy/difficult did you find it to identify/relate to the scenarios presented?
- What did you think of the alternative answers offered by the mobile application?
- How would you improve the content of sessions?

4**. What did you think about the number and length of the sessions?**

- How did you find the length of each individual session?
- What did you think about having 6 sessions in total?
- Would you change the number and length of sessions & if so, how?

**5. How did you find the experience of completing STOP on your own?**

- What were the advantages/advantages of this?
- What did you think about the 10min fortnightly check in calls with researcher?
- Could the app still be useful without these or are they essential?

6**. How did STOP compare to other treatments or therapies that you’ve received in the past?**

• What were the advantages/ disadvantages compared to others?

• Did you notice any impact (positive or negative) on your daily life of using STOP?

7. **Is there anything else that you would like to say about the mobile application and your experience of it before we end?**

STOP – Successful Treatment of Paranoia Version 1.0

Date: 20/11/2021

REC Reference: 303876

# Supplementary Methods SM2. Semi-structured interview topic guide on digital therapeutic alliance – STOP (Successful Treatment of Paranoia) app users.

**Topic guide for interview**

**Introduction**

Thank you for agreeing to take part in this interview. It should take up to 60 minutes, can I check you are happy to go ahead? As you know I’m hoping that the conversations we have will tell us more about how you experienced working the STOP App. I’m very grateful for your time today. I’ll explain how the interview will work and then you can ask any questions you have before we start. The interview will be recorded over Microsoft Teams, and it will also produce a transcription as we go along, this means a written recording of what we are saying. We keep all interviews secure and only members of the study team will have access to them. Once we have checked the transcripts for accuracy the recordings will be deleted, and transcripts will be anonymised. Everything you say is confidential. The only reason we would have to break confidentiality is if we are worried about your immediate safety. We would always talk to you first. If you would like to receive a copy of the results of the research let me know and I can email you that. We will use short quotes from some interviews, but we will always make sure to remove identifying information. After the study is completed, the anonymised transcripts will be stored in a data archive and may be used for further research. The interview will involve talking about your experience of working with the Stop App and how connected/what working relationship you had with that app. Throughout these questions consider the app’s features, for example its videos, activities, assessments, word tasks, questions**,** content, interface, menus, calendar, reminders, colour scheme, branding/logo, badges, trivia etc. You do not have to answer any questions you are uncomfortable with; you can stop the interview at any time and withdraw, take a break, or continue at another time. You can take your time with the questions; it can take time to reflect and that’s okay. I might sometimes ask if you’d like more time. At times I may interrupt you because there is something I want to know more about or am curious about. I hope that is okay. Do you have any questions?

**Was the STOP app helping you to do things that were working towards your mental health goals?**

- Did the exercises it asked you to do seem suited to your thinking patterns, behaviours and life experiences? How did that impact your connection/working relationship with the app?
- Were the things you were asked to do in the app manageable and meaningful in achieving your goals? How did the app do this? (For example, features, language, breaking things down)
- Did any features of the app encourage you to keep working towards your goals?

**Did you trust the app?**

- Was there anything the app did or said that made you feel this was an app with expertise?
- Did the app look and feel credible?
- How did this impact on your connection/working relationship with the app?

**Was the app able to motivate you to continue when you may have discontinued treatment?**

- How did the app do this (for example praising you, tracking your progress reminding you to do sessions?
- How did this impact your connection/working relationship with it?

**In this app there was not any opportunity in the app to connect with others with similar experiences or users of the app. For example, a forum or chat function where you could speak to other users of the app, read the anonymized stories of others who found the app helpful, or a leader board where you could compare your use of the app with others.**

- Is that something you found helpful for your connection or working relationship with the app?

**Have you had therapy before with a human being?**

**Why did you choose mental health apps instead of seeing therapist?**

- Was there anything the app was able to provide that a human therapist might not have?
- What do you think are the differences between your connection/working relationship with the therapist and this app?
- What do you think are the similarities between your connection/working relationship with the therapist and this app?
- Would you describe what you experienced with the app as a relationship/connection?
- Would you want to see a therapist and use the app simultaneously?
- How would you experience working alone with the app without a person being involved?

**Was there anything else you wanted to discuss today?**

- How did you find this interview?
- Any questions for me?
- I know there’s been some emotional things we have discussed. To help you transition to the next part of the day I want to check how you are doing. I’m going to send you the debrief form with some ideas of resources to look at following our call.
- I will send your voucher and debrief sheet
- Do they want to be informed of the results of the study?

# Supplementary Methods SM3. Semi-structured focus group topic guide on the perceived acceptability of STOP (Successful Treatment of Paranoia) – mental health workers.

**Introduction**

SET TEAMS TO RECORD AND TRANSCRIBE

Welcome and thanks everyone for agreeing to take part in this focus group. My name is [INSERT], and this is my colleague [INSERT]. I am [INSERT BRIEF SENTENCE HERE]. I will be leading today’s session. My most important role is to ask questions, keep us to time, and make sure you all have the chance to share your knowledge and experiences. My colleague [INSERT NAME] will help me with managing the discussion and will also be taking notes.

Now let's go around the group and have everyone briefly introduce themselves; please give your first name or preferred name and tell us your role.

Part of the evaluation of a new digital intervention for paranoia, called STOP, involves talking to stakeholders. We hope that conversations today will help us to understand your experiences of and ideas about the app. We will transcribe today’s conversation and analyse it along with the other interviews and focus groups we have done to find themes in what clinicians think about the app. We may also use anonymised quotes in write-ups of the data.

To recap, STOP was developed to help those experiencing paranoia (concerns that others or the world around them may cause harm). It aims to help people interpret ambiguous situations in a more neutral way by completing word puzzles and brain training activities, which are entirely delivered through a smartphone app. One session a week is completed independently, so at a time that works best for the user, for twelve weeks. The app includes several features to make it more interesting, including achievement badges and trivia facts.

We have a few ground rules to help us run the session today.

1. The discussion will last for about one hour. Let’s have one speaker at a time. Please give everyone the chance to express their opinion during the conversation. You can address each other if you like. We are only here to assist with the discussion.
2. There are no right or wrong answers. Everyone’s experiences and opinions are different, and we expect to hear a range of opinions and do not expect a consensus, just a discussion.
3. All the information we collect today is confidential. We will use the information you provide, but we will not identify you in anything we do related to this session.
4. We will record the session using Microsoft Teams as we want to capture everything you have to say. Is everyone okay with this?

SET TEAMS TO RECORD AND TRANSCRIBE

Does anyone have any questions before we begin?

Let’s get started...

| **Acceptability domain*** | **Question** | **Prompts** |
| --- | --- | --- |
| Affective attitude/ethicality | What do you think about the use of apps as part of treatment for mental illness? | - Have you previously used apps with clients to help with specific symptoms? - What aspects of your work with clients could apps help with? - Do you have any concerns about the integration of technology in mental healthcare? - Is there anything that would make you more confident about integrating technology into your practice? |
| Affective attitude/intervention coherence | As a potential prescriber, what are your thoughts on the STOP app? | - Was there anything you particularly liked or disliked about the app? - What did you think of the look and feel of the app? - Is there anything you might change about the app? |
| Burden/self-efficacy | The app is designed for use by anyone with self-reported symptoms of paranoia.  Do you think it could be useful to this group of people? | - Would you expect the app to reduce someone’s symptoms of paranoia? - Does the cognitive training provided by the app fit with your approach to treating paranoia? - Does the app seem accessible to clients? - How appropriate do you feel the mode of delivery is? - Is there anything that might help clients to stick with using the app? |
| Ethicality | Could you see the STOP app being appropriate for groups of people who aren’t always supported well by healthcare services? | - Are there any particular groups of service users, such as under-represented groups (e.g. minoritised ethnic groups, those involved in the criminal justice system), who you think may find engaging with the app difficult? - Are there any particular groups of service users who you think may find engaging with the app particularly useful? |
| Perceived effectiveness/opportunity costs | If shown to be effective, in what situations would you consider recommending the STOP app as part of treatment for paranoia? | - Could you envisage situations in which it might be unhelpful or inadvisable? - Is there any support you might need to help you make use of the app in your service? - Is there a point in a service user’s pathway where the app might be particularly appropriate? (e.g. waiting for therapy, clients who are recovered/who have residual symptoms, at risk mental state, adjunct to treatment) |
| STOP implementation study | Should the app be freely available to anyone, could you see it being useful to people with paranoia who aren’t currently using healthcare services? | - Why/why not? - Are there any possible benefits to making STOP available outside of healthcare services? - Are there any possible harms associated with making STOP available outside of healthcare services? - Is there anything that would help to make STOP accessible to people outside of healthcare services? - Are there any barriers to making STOP accessible to people outside of healthcare services? |

**Wrap up**

Thank you very much for taking part in this focus group. The information you have provided is very valuable.

As a thank you for your time, [researcher name] will send vouchers at the end of the recruitment period, which we expect to be January. If you would NOT like to receive a voucher, please let us know.

Are there any questions I can answer before we end the session?

Thank you again for your help. We really appreciate your time and your knowledge.

# Supplementary Methods SM4. Semi-structured focus interview topic guide on the perceived acceptability of STOP (Successful Treatment of Paranoia) – mental health workers.

**Introduction**

Thank you for agreeing to participate in the interview today. I am [INSERT BRIEF SENTENCE HERE]. I will be leading today’s session.

Please let me know what name I can use for you, and what your job role is.

Part of the evaluation of a new digital intervention for paranoia, called STOP, involves talking to stakeholders. We hope that conversations today will help us to understand your experiences of and ideas about the app. We will transcribe today’s discussion and analyse it along with the other interviews and focus groups we have done to find themes in what clinicians think about the app. We may also use anonymised quotes in write-ups of the data.

To recap, STOP was developed to help those experiencing paranoia (concerns that others or the world around them may cause harm). It aims to help people interpret ambiguous situations in a more neutral way by completing word puzzles and brain training activities, which are entirely delivered through a smartphone app. One session a week is completed independently, so at a time that works best for the user, for twelve weeks. The app includes several features to make it more interesting, including achievement badges and trivia facts.

1. The interview will last up to an hour.
2. There are no right or wrong answers. We expect to hear a range of opinions and are not looking for purely positive reviews of the app.
3. All the information we collect today is confidential. We will use the information you provide, but we will not identify you in anything we do related to this session.
4. We will record the session using Microsoft Teams as we want to capture everything you have to say. Is everyone okay with this?

SET TEAMS TO RECORD AND TRANSCRIBE

Any questions before we begin?

| **Acceptability domain*** | **Question** | **Prompts** |
| --- | --- | --- |
| Affective attitude/ethicality | What do you think about the use of apps as part of treatment for mental illness? | - Have you previously used apps with clients to help with specific symptoms? - What aspects of your work with clients could apps help with? - Do you have any concerns about the integration of technology in mental healthcare? - Is there anything that would make you more confident about integrating technology into your practice? |
| Affective attitude/intervention coherence | As a potential prescriber, what are your thoughts on the STOP app? | - Was there anything you particularly liked or disliked about the app? - What did you think of the look and feel of the app? - Is there anything you might change about the app? |
| Burden/self-efficacy | The app is designed for use by anyone with self-reported symptoms of paranoia.  Do you think it could be useful to this group of people? | - Would you expect the app to reduce someone’s symptoms of paranoia? - Does the cognitive training provided by the app fit with your approach to treating paranoia? - Does the app seem accessible to clients? - How appropriate do you feel the mode of delivery is? - Is there anything that might help clients to stick with using the app? |
| Ethicality | Could you see the STOP app being appropriate for groups of people who aren’t always supported well by healthcare services? | - Are there any particular groups of service users, such as under-represented groups (e.g. minoritised ethnic groups, those involved in the criminal justice system), who you think may find engaging with the app difficult? - Are there any particular groups of service users who you think may find engaging with the app particularly useful? |
| Perceived effectiveness/opportunity costs | If shown to be effective, in what situations would you consider recommending the STOP app as part of treatment for paranoia? | - Could you envisage situations in which it might be unhelpful or inadvisable? - Is there any support you might need to help you make use of the app in your service? - Is there a point in a service user’s pathway where the app might be particularly appropriate? (e.g. waiting for therapy, clients who are recovered/who have residual symptoms, at risk mental state, adjunct to treatment) |
| STOP implementation study | Should the app be freely available to anyone, could you see it being useful to people with paranoia who aren’t currently using healthcare services? | - Why/why not? - Are there any possible benefits to making STOP available outside of healthcare services? - Are there any possible harms associated with making STOP available outside of healthcare services? - Is there anything that would help to make STOP accessible to people outside of healthcare services? - Are there any barriers to making STOP accessible to people outside of healthcare services? |

**Wrap up**

Thank you very much for taking part in this interview. The information you have provided is very valuable.

As a thank you for your time, [researcher name] will send vouchers at the end of the recruitment period, which we expect to be January. If you would NOT like to receive a voucher, please let us know.

Are there any questions I can answer before we end the session?

Thank you again for your help. We really appreciate your time and your knowledge.
